# Supplementary material for: Complete genome sequence and comparative genomics of the golden pompano (Trachinotus ovatus) pathogen, Vibrio harveyi strain QT520
Source: PeerJ. 2017 Dec 8;5:e4127. doi: 10.7717/peerj.4127 (PMC5724406; doi:10.7717/peerj.4127)
Supplement: Table S1 — Accession numbers of 12 complete genome sequences of Vibrio sp. strains in the NCBI GeneBank database. [file peerj-05-4127-s001.doc]

-Supplementary Table1 Accession numbers of 12 complete genome sequences of *Vibrio sp.* strains in the NCBI GeneBank database

| Species | Strain | Chromosome | Accession numbers in GenBank |
| --- | --- | --- | --- |
| *V. harveyi* | ATCC 33843 (392 [MAV]) | chromosome 1 | CP009467.1 |
|  |  | chromosome 2 | CP009468.1 |
| *V. harveyi* | ATCC 43516 | chromosome 1 | CP014038.1 |
|  |  | chromosome 2 | CP014039.1 |
| *V. campbellii* | LMB29 | chromosome 1 | CP019293.1 |
|  |  | chromosome 2 | CP019294.1 |
| *V. campbellii* | 1114GL | chromosome 1 | CP019634.1 |
|  |  | chromosome 2 | CP019635.1 |
| *V. campbellii* | ATCC BAA-1116 | chromosome 1 | NC_022269.1 |
|  |  | chromosome 2 | NC_022270.1 |
| *V. alginolyticus* | ATCC 33787 | chromosome 1 | CP013484.1 |
|  |  | chromosome 2 | CP013485.1 |
| *V. alginolyticus* | ZJ-T | chromosome 1 | CP016224.1 |
|  |  | chromosome 2 | CP016225.1 |
| *V. parahaemolyticus* | FORC_004 | chromosome 1 | CP009847.1 |
|  |  | chromosome 2 | CP009848.1 |
| *V. parahaemolyticus* | ATCC 17802 | chromosome 1 | CP014046.1 |
|  |  | chromosome 2 | CP014047.1 |
| *V. natriegens* | CCUG 16373 | chromosome 1 | CP016349.1 |
|  |  | chromosome 2 | CP016350.1 |
| *V.coralliilyticus* | RE98 | chromosome 1 | CP009617.1 |
|  |  | chromosome 2 | CP009618.1 |
| *V. harveyi* | QT520 | chromosome 1 | CP018680.2 |
|  |  | chromosome 2 | CP018681.2 |
