# Supplementary material for: Complete genome sequence and comparative genomics of the golden pompano (Trachinotus ovatus) pathogen, Vibrio harveyi strain QT520
Source: PeerJ. 2017 Dec 8;5:e4127. doi: 10.7717/peerj.4127 (PMC5724406; doi:10.7717/peerj.4127)
Supplement: Table S2 — Genome and environmental features of V. harveyi QT520 according to the MIGS recommendations. [file peerj-05-4127-s002.doc]

-Supplementary Table 2 Genome and environmental features of *V. harveyi* QT520 according to the MIGS recommendations

| Item | Description |
| --- | --- |
| MIGS data |  |
| Investigation_type | Bacterial |
| Project_name | Genome sequencing of *V. harveyi* QT520 |
| Collected_by | Zhigang Tu |
| Collection_date | 2016 |
| Lat_lon | 19.9901N 109.8611E |
| Depth | 0-18m under water |
| Alt_elev | NA |
| Country | China |
| Environment | deep sea cage-cultured farm |
| Ref_biomaterial | NA |
| Biotic_relationship | Isolated from diseased fish |
| Trophic_level | Heterotroph |
| Rel_to_oxygen | Aerobic |
| Isol_growth_condt | TCBs, 30℃ |
| Sequencing_meth | PacBio |
| Num_replicons | NA |
| Assembly | Canu |
| Finishing_strategy | Complete |
| Annot_source | Prokka |
| Estimated_size | 6,130,289bp |
| Biome | ENVO:00000447 |
| Feature | ENVO:00000569 |
| Material | ENVO:00003875 |
| Geo_loc_name | China |
| Sample-material | Diseased fish *Trachinotus ovatus* |
| Source_mat_id | CP018680,CP018681, CP018682, CP018683, CP018684 |
| Genome assembly data |  |
| Assembly software | Canu, Gapcloser, GapFiller and PrInSeS-G |
| Assembly name | QT520 |
| Genome coverage | 227.76╳ |
| Sequencing technology | Illumina Miseq PE300 and PacBio RSII system |
