# Supplementary material for: Complete genome sequence and comparative genomics of the golden pompano (Trachinotus ovatus) pathogen, Vibrio harveyi strain QT520
Source: PeerJ. 2017 Dec 8;5:e4127. doi: 10.7717/peerj.4127 (PMC5724406; doi:10.7717/peerj.4127)
Supplement: Table S3 — Functional categorization of partial virulence-factor-encoding genes in strain QT520. [file peerj-05-4127-s003.doc]

-Supplementary Table 3 Functional categorization of partial virulence-factor-encoding genes in strain QT520.

| Virulence factor gene | Annotation | Chromosome/  Plasmid | Location(nt) |
| --- | --- | --- | --- |
| **Adherence** |  |  |  |
| acfA | Accessory colonization factor | ChromosomeII | 1136512-1137159 |
| cap8M^*^,  cpsA,  gmhA2,  kfiD,  kpsF,  neuB | enzyme  synthase  isomerase  dehydrogenase  isomerase  synthetase | ChromosomeI  ChromosomeI  ChromosomeI  ChromosomeI  ChromosomeI  ChromosomeI | 2954754-2955302  1758545-1759120  3125354-3125944  2957570-2958736  2187697-2188668  2913803-2914876 |
| IlpA | immunogenic lipoprotein A | ChromosomeI | 3432898-3433707 |
| ompU | outer membrane protein | ChromosomeI | 1964157-1965182 |
| **Iron uptake** |  |  |  |
| iutA | Aerobactin receptor IutA | Chromosome II | 775829-777967 |
| chuV,shuV,chuU | ATP-binding protein | Chromosome II | 348518-350337 |
| entB, entE, fepC | Ferrienterobactin ABC transporter ATPase | Chromosome I | 78297-79916 |
| feoB | Ferrous iron transporter B | Chromosome I | 27963-30239 |
| mbtA | Peptide synthetase | Chromosome I | 78297-79916 |
| ybtE | Inner membrane ABC-transporter | Chromosome I | 78297-79916 |
| **Motility** |  |  |  |
| cheY,  fleQ, fleR,  flgB,flgC,flgE,flgF,flgG, flgH,flgI,  flhA, flhB,  fliA, fliE fliG, fliI, fliM, fliN, fliP,fliQ,fliR, | Flagellin-related protein | Chromosome I  Chromosome I  Chromosome I  Chromosome II  Chromosome I  Chromosome I | 1701491-1701871  1721656-1725762  3507425-3514129  1700184-1705942  1705075-1708531  1701905-1721529  320203-325086 |
| **Sceretion system** |  |  |  |
| gspE, gspF, gspG | Type II secretion system | Chromosome I | 2833622-2836848 |
| bsaS, spaP | Type III secretion system | Chromosome I | 1716173-1717492  1645753-1647105 |
| iagB^*^ | Type III secretion system | Plasmid p2 | 64622-65065  23663-24106 |
| Type IV pili A^*^ | [T](http://www.mgc.ac.cn/cgi-bin/VFs/vfs.cgi?VFID=VF0156#Term-type%20IV%20secretion%20system)ype IV secretion system | Chromosome I | 2037875-2038333 |
| lpg2359,  lpg2936 | [T](http://www.mgc.ac.cn/cgi-bin/VFs/vfs.cgi?VFID=VF0156#Term-type%20IV%20secretion%20system)ype IV secretion system | Chromosome I  Chromosome I | 3085081-3085524  2126387-2127118 |
| clpB/vasG, clpB/vasG, clpB/vasG,  vasA, vasE,  vipA/mglA,vipB/mglB， | Type VI secretion system | Chromosome I  Chromosome II  Chromosome II  Chromosome II  Chromosome II | 736579-739185  720749-723304  1091268-1093946  1065404-1066729  1078512-1080502 |
| **Endotoxin** |  |  |  |
| manB^*^, bplL^*^ | enzyme | Chromosome I | 2950367-2957409 |
| bplB^*^ | enzyme | Chromosome II | 995263-995742 |
| **Toxin** |  |  |  |
| cyaB^*^ | Cyclolysin secretion ATP-binding protein | Plasmid p1 | 93664-95847 |
| hlyB^*^ | Hemolysin | Plasmid p1 | 97208-99376 |
| rtxA^*^ | RTX toxin | Plasmid p1 | 423-4103  102392-117676 |

Notes

^*^QT520 specific virulence-factor-encoding genes based on comparative genome analysis with ATCC 33843 (392 [MAV]) and ATCC 43516

-Supplementary Table 4 ANI values 0f 12 *Vibrio sp.* strains

| **OrthoANI** | **ATCC 33787** | **ZJ-T** | **1114GL** | **ATCC BAA-1116** | **LMB29** | **RE98** | **ATCC 33843 (392 [MAV])** | **ATCC 43516** | **QT520** | **CCUG 16373** | **ATCC 17802** | **FORC_004** |
| --- | --- | --- | --- | --- | --- | --- | --- | --- | --- | --- | --- | --- |
| **ATCC 33787** | 100% | 98.51% | 81.03% | 81.06% | 81.51% | 74.03% | 80.85% | 80.87% | 80.98% | 79.92% | 83.31% | 83.47% |
| **ZJ-T** |  | 100% | 81.22% | 81.32% | 81.08% | 74.04% | 81.00% | 80.93% | 81.01% | 79.88% | 83.48% | 83.48% |
| **1114GL** |  |  | 100% | 96.71% | 97.60% | 74.33% | 88.40% | 88.53% | 88.45% | 79.26% | 80.93% | 80.86% |
| **ATCC BAA-1116** |  |  |  | 100 % | 96.67% | 74.53% | 88.37% | 88.26% | 88.30% | 79.26% | 80.96% | 81.05% |
| **LMB29** |  |  |  |  | 100% | 74.36% | 88.32% | 88.23% | 88.19% | 79.15% | 80.65% | 80.56% |
| **RE98** |  |  |  |  |  | 100% | 74.15% | 74.34% | 74.18% | 74.12% | 74.12% | 74.11% |
| **ATCC 33843**  **(392 [MAV])** |  |  |  |  |  |  | 100% | 98.65% | 98.49% | 79.13% | 80.66% | 80.79% |
| **ATCC 43516** |  |  |  |  |  |  |  | 100% | 98.59% | 79.08% | 80.58% | 80.67% |
| **QT520** |  |  |  |  |  |  |  |  | 100% | 79.98% | 80.78% | 80.83% |
| **CCUG 16373** |  |  |  |  |  |  |  |  |  | 100% | 80.63% | 80.56% |
| **ATCC 17802** |  |  |  |  |  |  |  |  |  |  | 100% | 98.37% |
| **FORC_004** |  |  |  |  |  |  |  |  |  |  |  | 100% |
