# Supplementary material for: Complete genome sequence and comparative genomics of the golden pompano (Trachinotus ovatus) pathogen, Vibrio harveyi strain QT520
Source: PeerJ. 2017 Dec 8;5:e4127. doi: 10.7717/peerj.4127 (PMC5724406; doi:10.7717/peerj.4127)
Supplement: Table S4 — ANI values of 12 Vibrio sp. strains. [file peerj-05-4127-s004.doc]

-Supplementary Table 4 ANI values 0f 12 *Vibrio sp.* strains

| **OrthoANI** | **ATCC 33787** | **ZJ-T** | **1114GL** | **ATCC BAA-1116** | **LMB29** | **RE98** | **ATCC 33843 (392 [MAV])** | **ATCC 43516** | **QT520** | **CCUG 16373** | **ATCC 17802** | **FORC_004** |
| --- | --- | --- | --- | --- | --- | --- | --- | --- | --- | --- | --- | --- |
| **ATCC 33787** | 100% | 98.51% | 81.03% | 81.06% | 81.51% | 74.03% | 80.85% | 80.87% | 80.98% | 79.92% | 83.31% | 83.47% |
| **ZJ-T** |  | 100% | 81.22% | 81.32% | 81.08% | 74.04% | 81.00% | 80.93% | 81.01% | 79.88% | 83.48% | 83.48% |
| **1114GL** |  |  | 100% | 96.71% | 97.60% | 74.33% | 88.40% | 88.53% | 88.45% | 79.26% | 80.93% | 80.86% |
| **ATCC BAA-1116** |  |  |  | 100 % | 96.67% | 74.53% | 88.37% | 88.26% | 88.30% | 79.26% | 80.96% | 81.05% |
| **LMB29** |  |  |  |  | 100% | 74.36% | 88.32% | 88.23% | 88.19% | 79.15% | 80.65% | 80.56% |
| **RE98** |  |  |  |  |  | 100% | 74.15% | 74.34% | 74.18% | 74.12% | 74.12% | 74.11% |
| **ATCC 33843**  **(392 [MAV])** |  |  |  |  |  |  | 100% | 98.65% | 98.49% | 79.13% | 80.66% | 80.79% |
| **ATCC 43516** |  |  |  |  |  |  |  | 100% | 98.59% | 79.08% | 80.58% | 80.67% |
| **QT520** |  |  |  |  |  |  |  |  | 100% | 79.98% | 80.78% | 80.83% |
| **CCUG 16373** |  |  |  |  |  |  |  |  |  | 100% | 80.63% | 80.56% |
| **ATCC 17802** |  |  |  |  |  |  |  |  |  |  | 100% | 98.37% |
| **FORC_004** |  |  |  |  |  |  |  |  |  |  |  | 100% |
